# A new species and a new combination in *Phaeostemma* (Apocynaceae, Asclepiadoideae, Gonolobinae)

**DOI:** 10.3897/phytokeys.33.6453

**Published:** 2014-01-16

**Authors:** Gilberto Morillo, Alexander Krings

**Affiliations:** 1 Departamento de Botánica, Facultad de Ciencias Forestales y Ambientales, Universidad de Los Andes, Mérida 5101-A, Venezuela; 2 Herbarium, Department of Plant and Microbial Biology, North Carolina State University, Raleigh, NC 27695-7612, U.S.A.

**Keywords:** Climbing milkweeds, *Matelea*, Suriname, Venezuela

## Abstract

*Phaeostemma surinamensis* Morillo & Krings, **sp. nov.**, anew species of Apocynaceae (Asclepiadoideae, Gonolobinae) is described and illustrated, and the new combination *Phaeostemma fucata* (Woodson) Morillo & Krings, **comb. nov.**, is made. The new species, known only from a lowland wet forest of Suriname, seems to be closely related to *Phaeostemma fucata*, which is an endemic to Ptari-tepui, a sandstone mountain in the southeastern edge of the Venezuelan Guayana.

## Introduction

**Figure 1.**
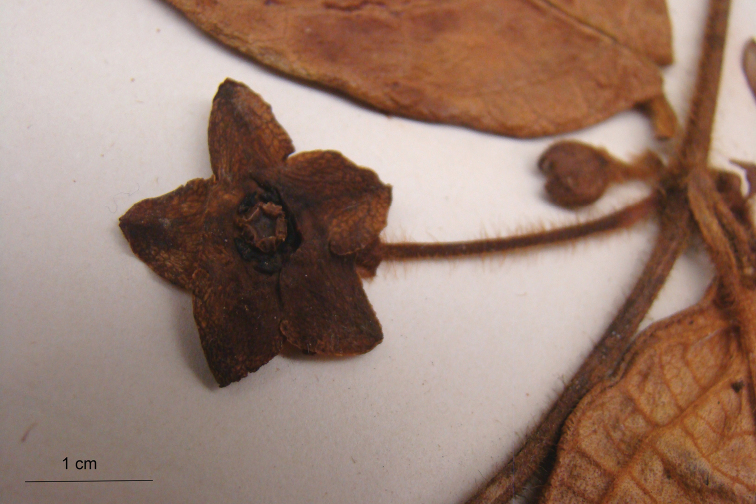
Flower of *Phaeostemma fucata* (from *Steyermark 59963*, MO).

**Figure 2.**
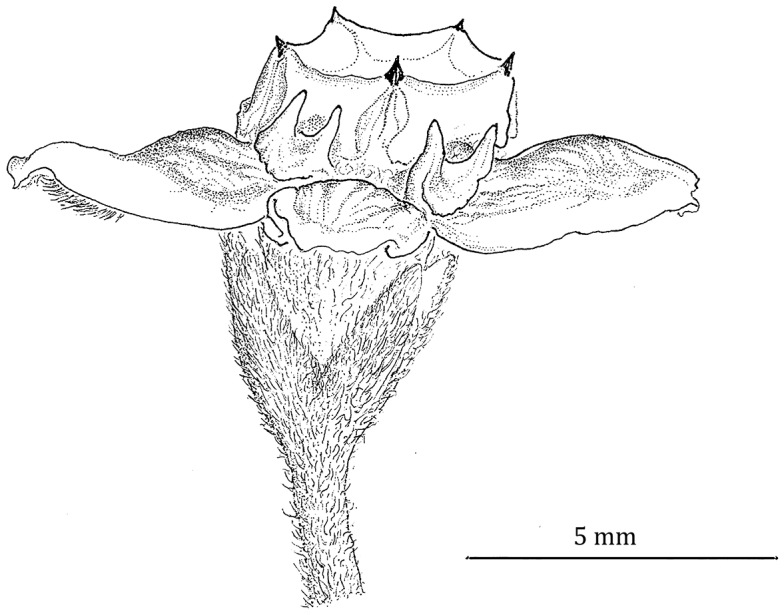
Flower of *Lachnostoma ecuadorensis* Morillo (*Homeier et al. 1174*, QCNE).

*Phaeostemma* E. Fourn. (Apocynaceae, Asclepiadoideae, Gonolobinae) is a South American genus of twining vines, distributed from southeastern Venezuela to Argentina. Heretofore, five names have been published in the genus — *Phaeostemma brandonianum* Silveira, *Phaeostemma glaziovii* E. Fourn., *Phaeostemma grandifolia* Rusby, *Phaeostemma riedelii* E. Fourn. and *Phaeostemma tigrina* Woodson—however, based on morphology, neither *Phaeostemma grandifolia* [syn. *Matelea dasytricha* (Schltr.) Woodson] nor *Phaeostemma tigrina* appear to belong to *Phaeostemma*. The latter species appear to belong to the Andean lineage *Lachnostoma* Kunth (Morillo 2012). Members of *Phaeostemma* s.l. are recognized by stems, leaves and inflorescences densely pubescent, with brown to yellowish-red medium to long (0.9–3 mm) eglandular trichomes, mixed with some shorter (0.5–1 mm) eglandular, multiseptate trichomes, and in some species short (0.15–0.4 mm) glandular trichomes, leaves membranous to coriaceous, often broadly ovate, ovate-elliptic, elliptic or widely oblong, medium to large in size (8–19.5 × 3.8–12 cm), bases shortly cordate, flowers large and broadly campanulate (corolla 23–37 mm in diameter; Fig. 1), corollas green to greenish-yellow, lobes ovate to deltate, sometimes reticulate, spreading, not ocellate, gynostegium stipitate, nectar chambers present, anthers subtriangular almost horizontal, radially prominent, pollinia narrowly or triangular pearshaped, and staminal corona fleshy, of 5 digitate lobes, partly adnate to the stipe and to the corolla tube. *Phaeostemma* is somewhat similar to *Lachnostoma*,but in *Lachnostoma*, the pubescence is usually shorter, with short to long (0.12–1.60 mm) eglandular trichomes, and rarely short (0.10–0.25 mm) glandular trichomes, and corollas usually smaller (12–20 mm in diameter; 26–34 mm in *Lachnostoma uribei* (Morillo) Morillo, inedit.), with lobes in natural position ovate-oblong to narrowly ovate-elliptic, longer than wide, partly due to recurved margins, in few species as long as wide, and bases narrowly campanulate or subtubular (Fig. 2). Species of *Lachnostoma* are known only from wet mountain forests (usually above 1400 m) from the Andes of Peru, Ecuador and Colombia to the Costal Range of northern Venezuela, whereas species of *Phaeostemma* are known mainly from Tropical Rain Forests or in Austral Forests with araucarias (mostly below 1000 m), from southeastern Guayana to northern Argentina.

*Lachnostoma* and *Phaeostemma* have been treated as nomenclatural synomyms of *Matelea* Aubl. sensu lato (Spellman and Morillo 1976; Morillo 1984; Fontella-Pereira et al. 1985), however, recent morphological studies (Morillo 2012), indicate that these genera differ from *Matelea* by the combination of dense, ubiquitous, long brown or yellowish-red pubescence in stems, leaves and inflorescences, corollas medium to large (12–37 mm in diameter), narrowly to broadly tubular, campanulate to subcampanulate, staminal corona segments (Cs) well-developed, connate to the base of the anthers, apically bifid or digitate, nectar chambers conspicuous, anthers radially prominent, with a concave outer face, retinacula broadly sagittate, and follicles (mature follicles unknown for *Phaeostemma*) ovate-oblong, with 5 wings and several conical projections. Members of *Matelea* s.s., in contrast, are recognized by stems glabrous to variously pubescent, pubescence in one or two lines, rarely ubiquitous, eglandular trichomes white or translucent, glandular capitate trichomes white, translucent, or with blackish capitula, corollas small to medium-size (less than 15 mm in diameter), usually rotate to subcampanulate, staminal corona segments (Cs) not distinct, usually appearing as ridges emanating from the central stipe, nectar chambers absent, retinacula narrowly sagittate or ovate-sagittate, and follicles narrowly ovate or fusiform, unwinged, costate, or 5-winged, conical projects absent.

On-going work for various regional projects, including the *Flora of the Guianas* and the Biological Diversity of the Guiana Shield, has resulted in the discovery of a new species of *Phaeostemma*: *Phaeostemma surinamensis* Morillo & Krings [initially misidentified as *Matelea glaziovii* (E. Fourn.) Morillo = *Phaeostemma glaziovii* E. Fourn (Morillo 1997)]. A more careful study of the known species of the genus resulted in the present recognition of its distinctness. The new species is morphologically similar to *Phaeostemma fucata* (Woodson) Morillo & Krings, an endemic to Ptari-tepui, a sandstone mountain in the Venezuelan Guayana. The new species is described and distinguished below, and the requisite new combination made.

## Taxonomic treatment

### Key to *Phaeostemma*

**Table:** 

1a	Adaxial corolla lobe surface glabrous	2
1b	Adaxial corolla lobe surface pubescent	4
2a	Trichomes of stems, midveins, and inflorescences eglandular; corolla lobes longer than wide, adaxially obscurely reticulate when dry, leaf blades membranous; southeastern Brazil	*Phaeostemma riedelii*
2b	Trichomes of stems, midveins, and inflorescences both eglandular and glandular; corolla lobes as long as wide or wider than long, adaxially conspicuously reticulate when dry, leaf blades membranous or coriaceous; Guayana and Suriname	3
3a	Leaf blades coriaceous, broadly ovate to ovate-elliptic, marginally revolute, calyx lobes 8.5 mm long; Guayana (Venezuela)	*Phaeostemma fucata*
3b	Leaf blades membranous, narrowly elliptic to oblanceolate-elliptic, marginally spreading; calyx lobes 5.5–6 mm long; Suriname	*Phaeostemma surinamensis*
4a	Leaves subcoriaceous, veins on adaxial surface distinctly impressed, blades mostly broadly elliptic to ovate	*Phaeostemma brandonianum*
4b	Leaves membranous, veins on adaxial surface not or only slightly impressed, blades mostly narrowly elliptic to slightly obovate	*Phaeostemma glaziovii*

#### Phaeostemma fucata

(Woodson) Morillo & Krings comb. nov.

urn:lsid:ipni.org:names:77135536-1

http://species-id.net/wiki/Phaeostemma_fucata

Figure 1

Matelea fucata Woodson, Fieldiana, Bot. ser. 28(3): 510. 1953.

##### Type.

VENEZUELA. Bolivar, Ptari-tepui, densely forested slopes overlying sandstone, alt. 1800 m, 8 Nov 1944, *J. Steyermark 59963* (Holotype: MO!; Isotypes: F!, NY!, VEN!).

#### Phaeostemma surinamensis

Morillo & Krings sp. nov.

urn:lsid:ipni.org:names:77135538-1

http://species-id.net/wiki/Phaeostemma_surinamensis

**Figure 3.**
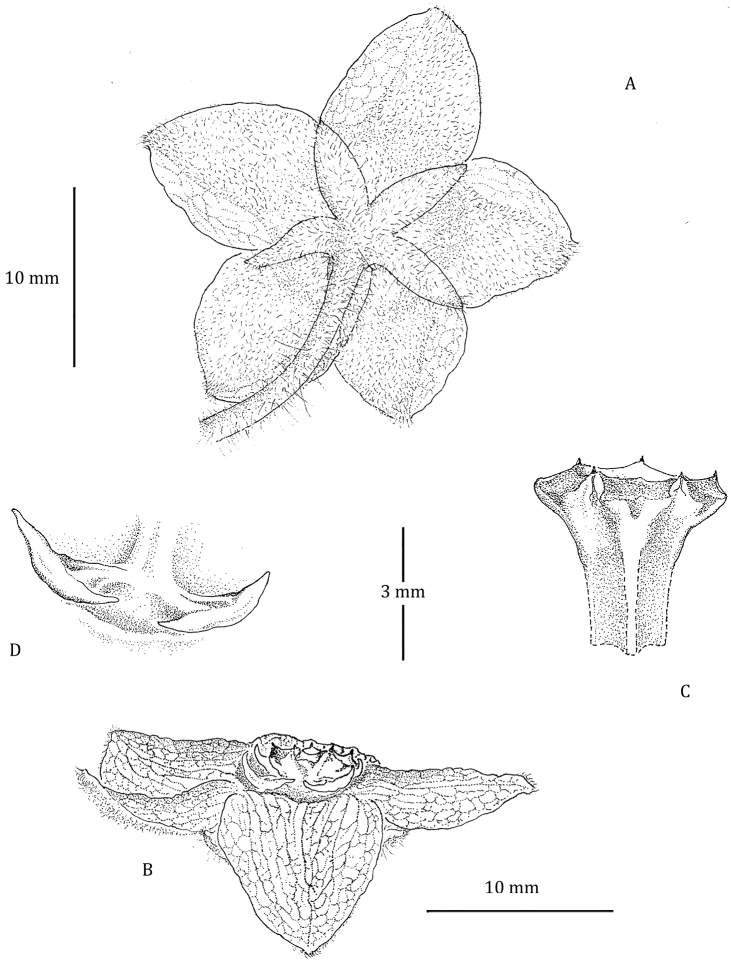
Flower of *Phaeostemma surinamensis*. **A** Abaxial view showing calyx and abaxial corolla lobes **B** Side view showing adaxial corolla surface and gynostegial corona **C** Style-head **D** Staminal corona (Cs). Based on *Lindeman et al. 654* (U).

Figures 3 [Fig F4] [Fig F5] –6

A new species of *Phaeostemma* E. Fourn., morphologically similar to *Phaeostemma fucata* (Woodson) Morillo & Krings, but differing from the latter among other characters, by leaf blades thinner, membranous, narrowly elliptic to oblanceolate-elliptic, marginally spreading (vs. coriaceous, broadly ovate to ovate-elliptic, marginally revolute in *Phaeostemma fucata*), calyx lobes 5.5–6 mm long (vs. 8.5 mm long in *Phaeostemma fucata*), staminal corona lobes (Cs) 3–3.2 mm wide, narrower at lateral extremes (vs. 2.3 mm wide at apex, somewhat obtuse at lateral extremes [projections] in *Phaeostemma fucata*), and pollinia longer, 1–1.15 mm (vs. ca. 0.8 mm long in *Phaeostemma fucata*).

##### Type.

**SURINAME.** Lely Mts., SW plateaus covered by ferrobauxite, in secondary vegetation, at end of airstrip, alt. 550–710 m, 1 Oct 1975, *J.C. Lindeman, A.L. Stoffers, A.R.A. Górts-van Rijn & M.J. Jansen-Jacobs 654* (Holotype: U!; Isotype: MO!).

##### Description.

*Vine*, woody, slender. *Stems* densely pubescent, pubescence mixed eglandular and glandular trichomes, eglandular trichomes ubiquitous reddish-brown, stiff, usually spreading, multiseptate, 1.2–2.3 mm long, glandular trichomes spreading, scarce in mature stems, 0.2–0.3 mm long. *Leaf* blades membranous, narrowly elliptic to oblanceolate-elliptic, 10.2–15.0 × 3.8–5.8 cm (1.6–1.8 cm wide at base), apex short acute, base narrowly and shortly cordate, trichomes of surfaces, veins, and margins mixed, yellowish-red, adaxial surface strigose, eglandular multicelled trichomes antrorse, curved to subappressed 0.7–1.3 mm long, glandular trichomes 3–4-celled, spreading, 0.15–0.2 mm long, present mainly on midvein, abaxial surface hispid or hispidulous, eglandular multicelled trichomes erect, 0.25–0.7 mm long, glandular trichomes spreading on the midvein, 0.1–0.2 mm long, midvein adaxially sulcate, abaxially prominent, lateral veins in 6–7 pairs, slightly to strongly prominent; colleters 2, digitate-cylindric, 2.8–3.3 mm long; petioles 1.1–1.7 cm long, densely pubescent, pubescence ubiquitous, eglandular multicelled trichomes spreading, 2.8–3.3 mm long, glandular trichomes 3–4-celled, spreading, 0.1–0.2 mm long. *Inflorescence* racemiform, 2–4-flowered, 1(–2) flowers open at a time; peduncles 3–5 mm long, sparsely to moderately pubescent, pubescence ubiquitous, eglandular trichomes spreading, 1.2–2 mm long, glandular trichomes 3–4-celled, scarce, spreading, 0.2–0.3 mm long, rachis with scars, 3–4 mm long, bracts oblong, 1.8–2 mm long, abaxially pubescent, eglandular trichomes multicelled, 0.3–0.4 mm long, pedicels 24–33 mm long, densely pubescent, pubescence ubiquitous, eglandular trichomes spreading, 0.9–2.5 mm long, glandular trichomes 3–4-celled, spreading, 0.15–0.3 mm long. *Calyx* 8.5 mm long, lobes green, dark red at apex, oblong-elliptic, 5.5–6 × 2.4–2.6 mm, apex obtuse, margins entire, adaxial surface mostly glabrous, except few trichomes at apex, abaxial surface densely pubescent, eglandular multicelled trichomes spreading or antrorse, 0.9–1.5 mm long, glandular trichomes 2–3-celled, spreading, 0.1–0.3 mm long, colleters 1 per sinus. *Corolla* pale green, with green vein network (fide collectoris), broadly subcampanulate, tube 5.5–5.8 mm long, up to 7 mm wide, sparsely short pubescent on the abaxial side, lobes imbricate in bud, deltoid, spreading, 8–9 × 9–10 mm, adaxial surface glabrous, abaxial surface densely pubescent, eglandular trichomes reddish-brown curved, antrorse, 0.5–1.5 mm long, glandular trichomes 3–4-celled, scarce, 0.15–0.25 mm long, apices obtuse-emarginate, margins entire. *Gynostegium* stipitate, style-head green yellow, somewhat concave, 4.3–4.5 mm in diameter, stipe 2.5 mm long, terminal style-head appendage absent. *Corona* gynostegial, fleshy, of 5 staminal (Cs) apically bifid segments fused to the corolla tube for most of its length, free in the upper 1.5–1.6 mm, apical area bifurcate, dorsally flattened, 3–3.2 mm wide at apex; anthers subtriangular, 1.9–2.1 mm wide between wings; nectar chambers ca. 2.5 × 2.6 mm. *Pollinarium*: corpuscula narrowly obovate-sagittate, ca. 0.5 × 0.33 mm long, caudicles ca. 0.22 mm long, pollinia triangular-pyriform, 1.0–1.15 × 0.6 mm. *Follicles* in very immature state, ca. 2 cm long, apparently 5-costate, densely glandular-pubescent, eglandular trichomes not seen, glandular trichomes 2–3-celled, 0.1–0.15 mm long. *Seeds* unknown.

**Figure 4. F4:**
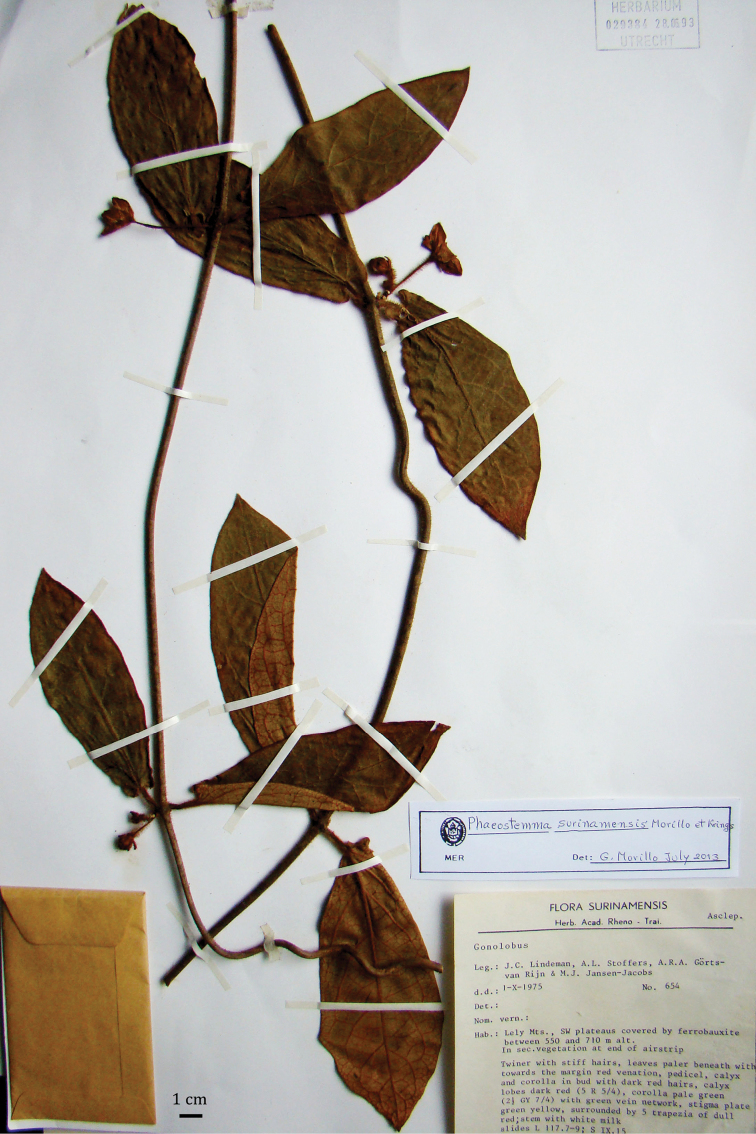
Holotype of *Phaeostemma surinamensis* (*Lindeman et al. 654*, U).

**Figure 5. F5:**
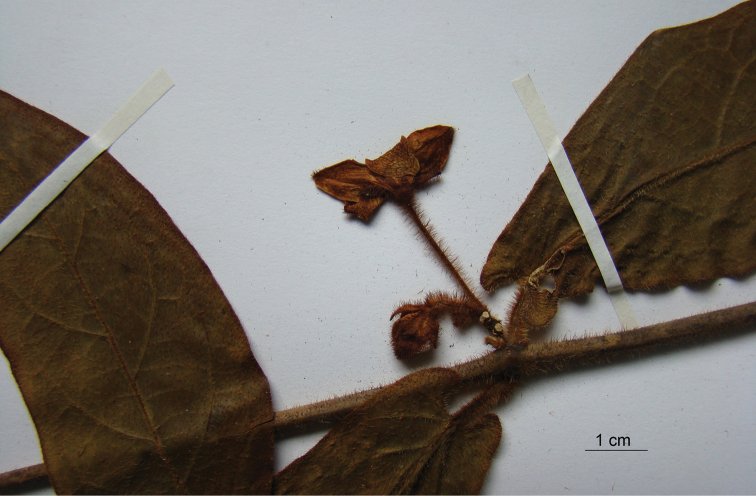
Detail of flower (side view) of *Phaeostemma surinamensis* from holotype (*Lindeman et al. 654*, U).

**Figure 6.**
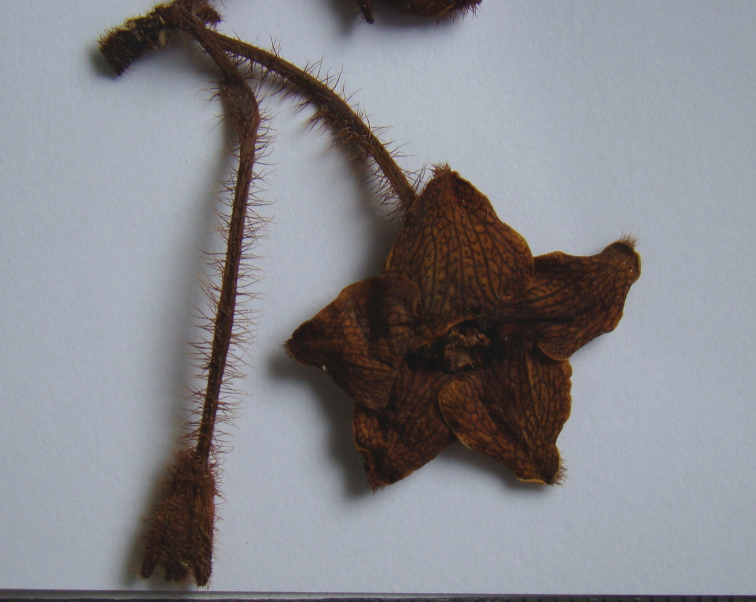
Detail of flower (adaxial view) of *Phaeostemma surinamensis* from holotype (*Lindeman et al. 654*, U).

##### Distribution and ecology.

Growing on a plateau covered with ferrobauxite rock. Endemic to Suriname, in tropical rain forests 500 to 710 m.

##### Phenology.

Collected in flower in October.

##### Conservation status.

Currently, very little is known regarding the status of this species.

### Excluded names

*Phaeostemma grandifolia* Rusby, Descr. S. Am. Pl. 101. 1920. = *Matelea dasytricha* (Schltr.) Fontella, Bradea 4 (9): 55. 1984 (syn. *Gonolobus dasytrichus* Schltr., Notizbl. Königl. Bot. Gart. Berlin 6(55): 177. 1914.)

*Phaeostemma tigrina* Woodson, Ann. Missouri Bot. Gard. 18: 560. 1931. = *Lachnostoma tigrinum* Kunth, Nov. Gen. Sp. (quarto ed.) 3: 199, t. 232. 1818[1819] (syn. *Matelea humboldtiana* Spellman & Morillo, Phytologia 34(2): 152. 1976.)

## Supplementary Material

XML Treatment for Phaeostemma fucata

XML Treatment for Phaeostemma surinamensis
